# Glutaminolysis and CD4^+^ T-cell metabolism in autoimmunity: From pathogenesis to therapy prospects

**DOI:** 10.3389/fimmu.2022.986847

**Published:** 2022-09-23

**Authors:** Xiaojin Feng, Xue Li, Na Liu, Ningning Hou, Xiaodong Sun, Yongping Liu

**Affiliations:** ^1^ Department of Endocrinology and Metabolism, Affiliated Hospital of Weifang Medical University, Weifang, China; ^2^ Department of Clinical Research Center, Affiliated Hospital of Weifang Medical University, Weifang, China; ^3^ Department of Pathology, Affiliated Hospital of Weifang Medical University, Weifang, China

**Keywords:** glutamine, glutaminolysis, CD4^+^ T cells, autoimmune diseases, immune response

## Abstract

The recent increase in the pathogenesis of autoimmune diseases revealed the critical role of T cells. Investigation into immunometabolism has drawn attention to metabolic processes other than glycometabolism. In rapidly dividing immune cells, including T lymphocytes, the consumption of glutamine is similar to or higher than that of glucose even though glucose is abundant. In addition to contributing to many processes critical for cellular integrity and function, glutamine, as the most abundant amino acid, was recently regarded as an immunomodulatory nutrient. A better understanding of the biological regulation of glutaminolysis in T cells will provide a new perspective for the treatment of autoimmune diseases. In this review, we summarized the current knowledge of glutamine catabolism in CD4^+^ T-cell subsets of autoimmunity. We also focused on potential treatments targeting glutaminolysis in patients with autoimmune diseases. Knowledge of immunometabolism is constantly evolving, and glutamine metabolism may be a potential therapeutic target for autoimmune disease therapy.

## 1 Introduction

Autoimmune diseases are chronic immune conditions caused by dysfunctional lymphocytes and excessive autoantibodies. The aberrant immune system is not able to distinguish its own components from non-self components, leading to dysfunction of tissues and organs (1, 2). Even though the pathogenesis of autoimmune diseases has not been fully illuminated, qualitative or quantitative defects of T cells are undisputedly a central part of the process (3).

Recent investigations into immunometabolism have drawn attention to metabolic processes other than glycometabolism that are involved in the regulation of the immune system (4). As the most abundant amino acid in human blood, glutamine (Gln) serves as a substrate in many critical biosynthetic processes (5). In addition to contributing to many processes critical for cellular integrity and function, Gln was recently identified as an immunomodulatory nutrient (6, 7). In rapidly dividing immune cells, including lymphocytes, the consumption of Gln is similar to or higher than that of glucose even when glucose is abundant (8). Glutaminolysis has been clearly revealed to be an energy supplier for T cells (9). Even though previous studies have revealed increased Gln catabolism during the differentiation of several T-cell subsets (10), the immunopathogenesis of metabolic enzymes remains poorly understood. Thus, a better understanding of the biological regulation of glutaminolysis in T cells, especially CD4^+^ T cells, will provide a new perspective for the treatment of autoimmune diseases.

In the present study, we will summarize the current understanding of glutamine catabolism in CD4^+^ T-cell subsets of autoimmunity, and we will also discuss potential treatments targeting glutaminolysis in patients with autoimmune diseases.

## 2 Glutamine metabolism

Gln is the most abundant amino acid in the human body (11). The concentration of Gln is from 10 to 100 times higher than that of other amino acids and it accounts for approximately 40% to 60% of the total amino acids in plasma and tissues (11). Gln is mainly concentrated in the liver and skeletal muscle tissue (11). In a healthy body, Gln levels in plasma and tissues are stable, and the maintenance of the Gln concentration depends on the balance between the supply and its consumption by organs and tissues (11). In the catabolic state, Gln is deficient, and immune function is impaired with a decreased proliferation capacity of immune cells. Then, Gln in muscle will be mobilized and turn into a conditionally essential amino acid to provide energy for the organism (12, 13).

**Figure 1:**
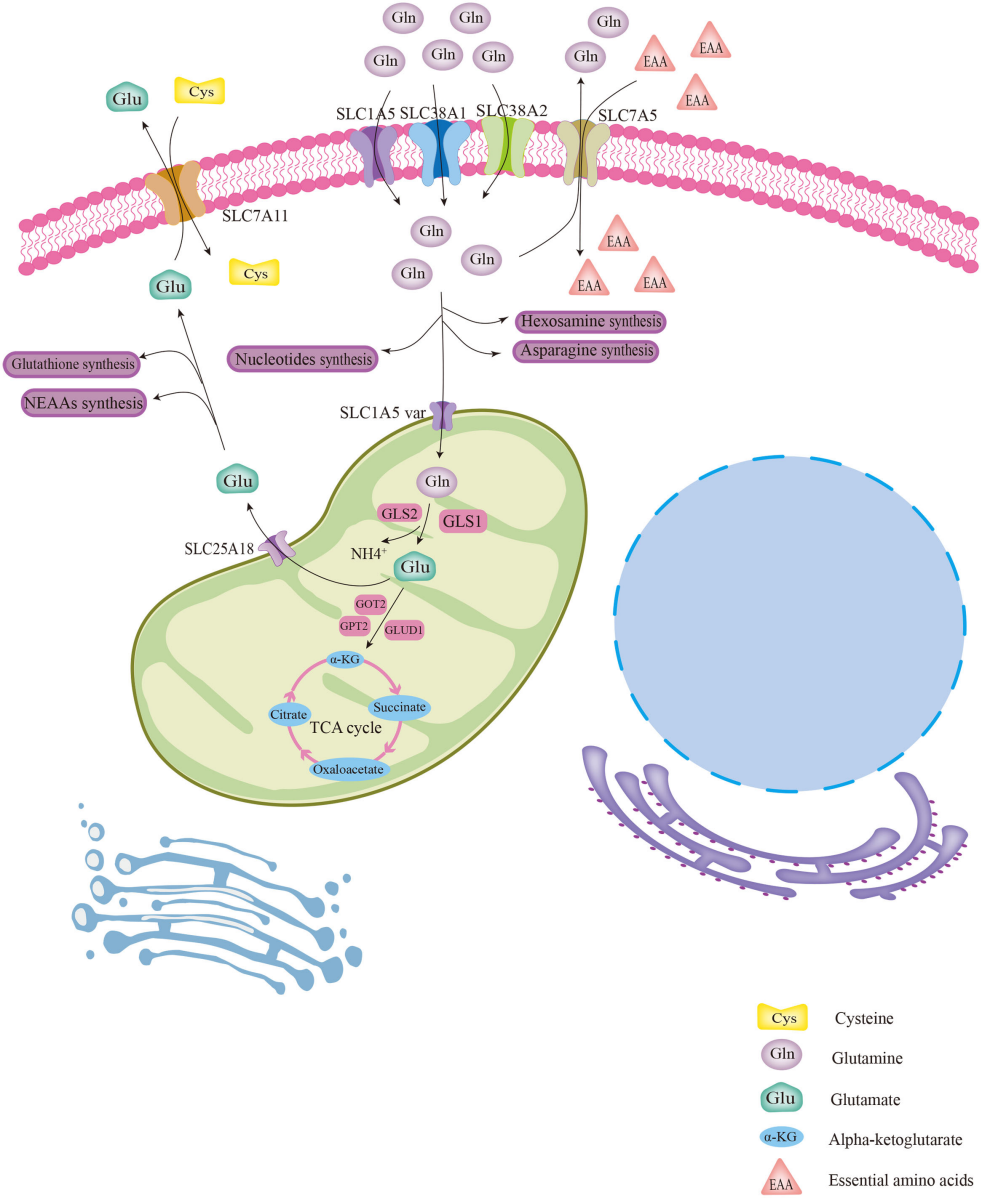
The biological process of glutamine metabolism in cells. Glutamine enters the cytoplasm with the help of several membrane transport proteins and is used for the synthesis of other biomolecules, such as hexosamine, nucleotides, and asparagine. Part of intracellular glutamine (Gln) is transported into the mitochondrial matrix *via* the SLC1A5 variant and subsequently converted to glutamate (Glu) with the help of GLS. Then, by catalysis of GLUD1 or several aminotransferases, Glu was converted to α-KG, which is involved in the TCA cycle. Glu was also transported out of mitochondria for the synthesis of glutathione and NEAAs *via* the transporter protein SLC25A18. The cytoplasmic Gln and Glu can be exchanged with extracellular EAAs and cystine, respectively, *via* transporter proteins.

Gln is implicated in a variety of roles in cellular metabolism. Generally, Gln is transported into the cytoplasm or out of cells with the help of membrane transporters such as SLC1A5, SLC38A1, and SLC38A2 (**Figure 1**) (14). Intracellular Gln can also be imported by exchange with essential amino acids (EAA) *via* the surface transporter protein SLC7A5 (**Figure 1**) (15). Intracellular Gln supports the synthesis of molecular organisms such as hexosamine, nucleotides, and asparagine in the cytoplasm (**Figure 1**) (14). Cytoplasmic Gln is transported into the mitochondria *via* the SLC1A5 variant (**Figure 1**) (14). Next, glutaminases (GLSs), including glutaminase 1 (GLS1), glutaminase 2 (GLS2) and GAC (a splicing isoform of GLS1), convert Gln to glutamate (Glu) in the mitochondria, releasing ammonium ions (**Figure 1**) (14). Most of the Glu in the mitochondria is later transformed to alpha-ketoglutarate (α-KG) by the action of glutamate dehydrogenase 1 (GLUD1) or glutamic-pyruvic transaminase 2 (GPT2) and glutamic-oxaloacetic transaminase 2 (GOT2) (**Figure 1**) (14). Then, α-KG in mitochondria is involved in the oxidative phosphorylation pathway or the reductive carboxylation pathway in the TCA cycle (**Figure 1**) (14). Part of Glu is exported out of the mitochondria *via* the transporter protein SLC25A18, and then the Glu in the cytosol is involved in the biosynthesis of glutathione and nonessential amino acids (NEAAs) (**Figure 1**) (14). Part of the Glu in the cytoplasm is used to exchange for extracellular cysteine with the help of the cell membrane transporter protein SLC7A11 (**Figure 1**) (14, 15).

## 3 The key role of glutaminolysis in the immune system and T cells

Gln has been reported to be essential for the biological process of proliferation and differentiation and survival of immune cells, protecting from various pathogens (13). Under catabolic conditions, the demands for Gln of immune cells increase dramatically. In fact, a high rate of Gln utilization by immune cells was discovered starting in the early/mid 1980s (13). Since then, the importance of Gln metabolism for immune cell function has become apparent (13).

### 3.1 Innate immune system

The immune system is made up of the innate immune system and the acquired immune system (16). As an initial line of defense to defend against pathogenic bacteria, the innate immune system is consist of antimicrobial peptides, the complement system, and immune cells such as macrophages and dendritic cells (16). When the innate immune system encounters a pathogen, it reacts immediately to kill the pathogen or remove it from the host, without pre-adaptation from the environment (16).

#### 3.1.1 Glutamine and skin

As the first line of defense of the organism, intact skin is a primary physical barrier against the invasion of harmful organisms (17). It has been found that large amounts of glutamine synthetase (GS) are stored in epidermal keratin-forming cells in human and rat skin sections. GS acts as a precursor for the synthesis of several biologically active compounds, such as purines, pyrimidines, and amino sugars, in rapidly dividing cells (18). The regulation of the immune state of the skin is strongly dependent on epidermal keratin-forming cells (19). Several studies have found that GLS1-mediated Gln catabolism promotes excessive proliferation and chemotaxis of psoriatic keratin-forming cells (19). With these findings, it is clear that the skin immune system is closely linked to Gln catabolism.

#### 3.1.2 Glutamine and intestinal immunity

As a complex, multicellular organ, the intestine performs numerous physiological functions that are critical in enteral nutrition (20). The intestinal mucosa contains a variety of immune cells in addition to enterocytes with absorptive functions. The intestinal innate immune system serves as the host’s initial line of defense against intestinal pathogens and is currently considered the largest immune organ in the body (21). Recent studies suggest that Gln might be a dietary component necessary to maintain intestinal mucosal metabolism, structure, and function during injury or stress (22). The ability of the intestinal mucosa to metabolize Gln probably acquires greater prominence in catabolic disease states when Gln depletion might be severe and oral nutrition might be interrupted by the severity of the disease (22). The administration of Gln *via* the rectal route in a rat colitis model downregulated the expression of regulatory inflammatory transcription factors (STAT1 and STAT5) and inflammatory factors, which alleviated the inflammatory effects of colitis (23). Clinically low plasma and intracellular Gln concentrations and decreased mucosal glutaminase activity in Crohn’s disease patients indicated that Gln metabolism was impaired therein, from which it was hypothesized that Gln supplementation would improve the clinical manifestations of Crohn’s disease (24). The application of Gln in a mouse model of sepsis prevented apoptosis and the intestinal inflammatory response of intestinal intraepithelial lymphocyte (IEL) γδT cells and downregulated inflammation-related mediator genes expressed by IEL γδT cells, thereby reducing the extent of sepsis-induced intestinal epithelial injury (25, 26). Parenteral supplementation with Gln combined with enteral nutrition was found to improve intestinal immune function by reducing apoptosis of Peyer’s patches (PPs), one of the lymphoid tissues associated with the gut (GALT), and to increase the number of PPs and IgA plasma cells in the lamina propria of the gut (27).

### 3.2 Acquired immune system: T cell implication

The acquired immune system is the most important component of the immune system, removing specific pathogens that infect the body, which is mainly mediated by T cells (16). T cells are the primary component of the acquired immune system. They convey specific antigen recognition receptors and function as highly specialized effectors capable of forming long-term immune memory. Naïve T cells are reorganized in primary immune organs. Then, maturation at the primary site is stimulated by pathogens, and the T-cell antigen receptor (TCR) is expressed. After maturing at the primary site, T cells are transported to secondary immune organs, such as lymph nodes, spleen, appendix, tonsils, and adenoids, where T cells activate and differentiate into subpopulations of cells that perform immune functions (28). T lymphocytes can be divided functionally into two main subsets: CD4^+^ T helper cells (Th) and CD8^+^ cytotoxic T lymphocytes (CTL) (29). CD4^+^ T cells are indirectly involved in the clearance of infections by regulating the activity of other immune cells such as macrophages, neutrophils, B cells and CTL. Although the CD4^+^ T cells is essential for clearance of infections, dysregulation may also lead to pathological conditions such as autoimmune diseases (30).Current investigations have revealed that the activation of T cells requires Gln and/or Gln metabolism, and the activation of T cells may also promote the uptake and metabolism of Gln. Gln is irreplaceable for the immune system because of its important function in the different stages of differentiation of immune cells and the high utilization of Gln throughout the immune system (31).

#### 3.2.1 Glutamine and thymus

As the principal lymphoid organ, thymus is critical for the production and maturation of T cells (32). Rat thymocytes showed high efficiency of Gln utilization during both resting and proliferation, and the maximal activities of glutaminase, Glu dehydrogenase, and aspartate aminotransferase were significantly increased in proliferating thymic cells (33). The utilization rate of Gln increased and the carbon and nitrogen contents of glutaminolysis completely recycled (34). Intestinal supplementation with α-KG may ameliorate thymic degeneration induced by endotoxemia in rats and restore muscle Gln levels with improved immune function (35). Parenteral Gln supplementation combined with enteral nutrition increased plasma and tissue Gln concentrations by upregulating the expression of heat shock protein 90 (Hsp90) and attenuated apoptosis in the lymphoid organ spleen and circulating lymphocytes, ultimately enhancing immune function and improving survival in severely burned rats (36).

#### 3.2.2 Glutamine and spleen

The spleen, which combines the immune dynamic balance of the innate and acquired immune systems, is a vital organ and is primarily responsible for the immune surveillance of the blood (37, 38). T lymphocytes are present throughout the spleen as key effectors of the acquired immune system, where their localization varies with their activation status and organization by the expression of cell surface receptors and chemotactic gradients (39). Whether rat, mouse, and human splenic lymphocytes were resting or following mitogenic stimulation, Gln was essential for lymphocyte proliferation and provided the essential signals required for cell proliferation (40). Parenteral supplementation with Gln combined with enteral nutrition reduced the release of inflammatory cytokines, attenuated apoptosis in the lymphoid organ spleen, enhanced immune function, and improved survival in septic rats (41). The percentage of blood T-lymphocyte and CD4^+^ T-cell populations was maintained in mice pretreated with Gln in the sepsis group, and the expression of the antiapoptotic Bcl-2 gene was more pronounced and increased in splenic CD4^+^ T cells. In sepsis mouse models pretreated with Gln, the activation of CD4^+^ T cells was in equilibrium, and the expression of the anti-apoptotic protein Bcl-2 was more pronounced (42). Gln administration significantly inhibited acute graft-versus-host disease (aGVHD)-induced inflammation and tissue damage in the spleen (43).

#### 3.2.3 The biological functions of glutaminolysis in CD4^+^ T cells

T lymphocytes are a vital population of immune cells involved in immune regulation and are a key component of the adaptive immune system (44). Presently, most studies focused on the differentiation of CD4^+^ T cells, especially the differentiation of subsets of CD4^+^ lymphocytes. In a specific cytokine environment, CD4^+^ T cells stimulated by antigens activate and differentiate into different subsets of helper T (Th) cells, including Th1, Th2, Th9, Th17, Th22, T regulatory (Treg), and T follicular helper (Tfh) cells. These subpopulations are determined by the signaling patterns they receive during their initial interaction with the antigen (45).

**Figure 2:**
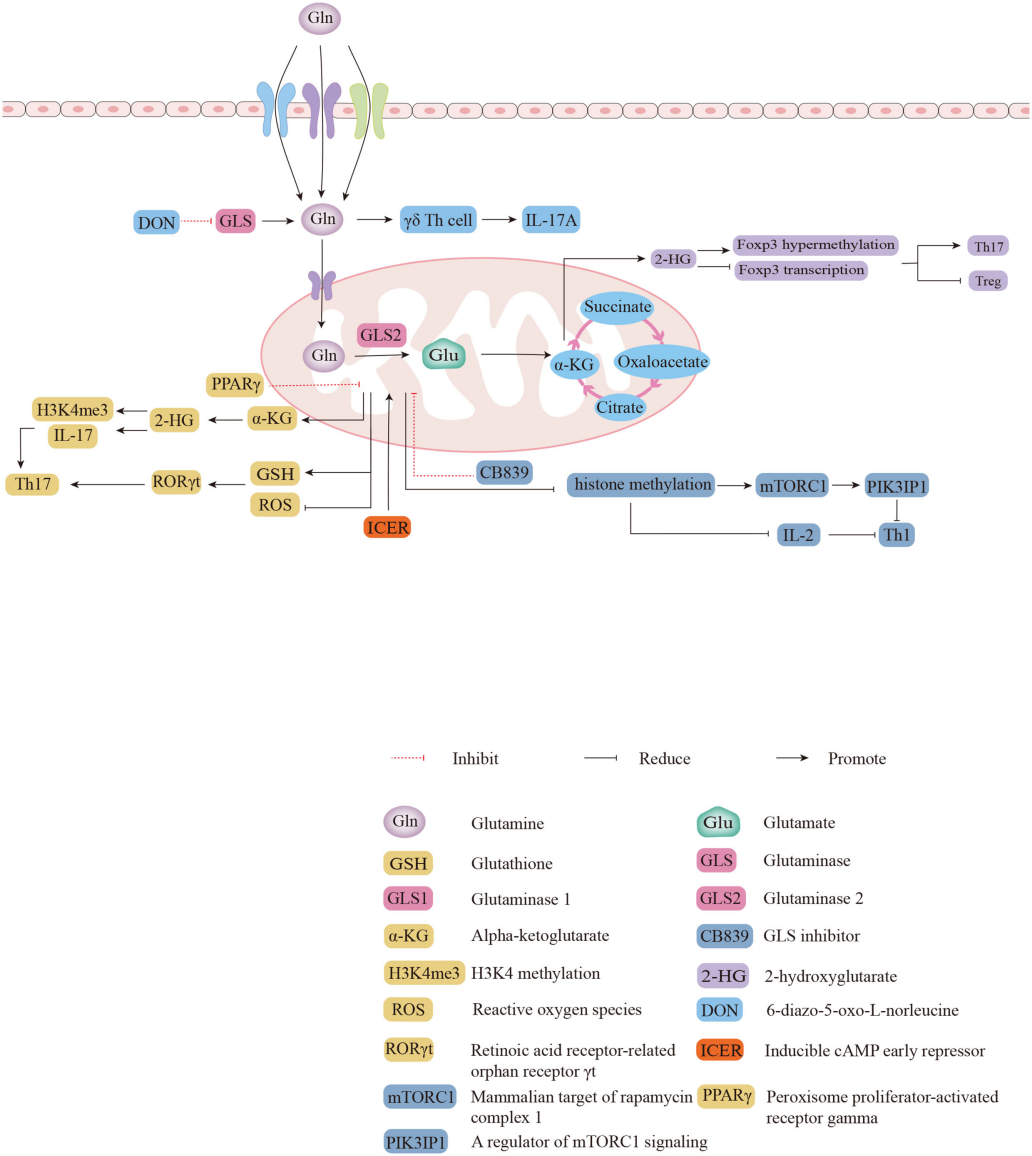
The biological functions of glutaminolysis in differentiations of different subsets of CD4^+^ T cells. 2-HG triggers hypermethylation and represses transcription of the Foxp3 gene to regulate Th17/Treg homeostasis. The expression of IL-17A was reduced in GLS inhibitor DON-treated γδ T cells. Direct binding of ICER to GLS1 promotes GLS1 expression and increases the oxygen consumption rate (OCR) of Th17 cells. PPARγ agonists inhibit Th17 responses by eliminating GLS1 through two pathways. Inhibition of mTORC1 and IL-2 signaling by GLS1 with CB839 attenuates Th1 differentiation.

Gln deficiency completely eliminated the proliferation of human CD4^+^ T cells, and inhibition of GLS reduced the proliferation index of human CD4^+^ T cells during the process of CD3/CD28 signaling activation (46). A previous study reported that α-KG, the metabolite of Glu, is more abundant in Th17 cells than in Treg cells, suggesting that glutaminolysis may be more active in Th17 cells (47).Further mechanistic findings revealed that 2-hydroxyglutarate (2-HG), a direct product of error-prone dehydrogenase activity on α-KG, could trigger hypermethylation of the Foxp3 gene and inhibit Foxp3 transcription, thereby inhibiting the differentiation of Treg cells and regulating Th17/Treg homeostasis through epigenetic mechanisms (**Figure 2**) (48). The differentiation of activated naïve CD4^+^ T cells into Th17 cells is severely impaired in glutamine-free medium, but Treg cell formation is normal and even appears to be enhanced in expression (10). Both Th17 and Th1 cells in cultures supplemented with Gln exhibit a dose-dependent induction, but the addition of Gln has only a mild effect on the production of Treg cells (49). The addition of excess Gln reversed the defective differentiation of SLC1A5^-/-^ T cells into Th17 cells (49). Gln can also promote the production of IL-17A from γδ T cells *via* nucleotide synthesis and the nitrogen-derived action of α-KG (**Figure 2**) (50). Treatment of γδ T cells with the GLS inhibitor 6-diazo-5-oxo-L-norleucine (DON) resulted in a significant decrease in the percentage of IL-17A^+^ γδ Th cells and led to a reduction in IL-17A expression in γδ T cells by more than half (50). Peroxisome proliferator-activated receptor gamma (PPARγ) agonists inhibit Th17-cell production by eliminating GLS1, reducing the levels of the downstream metabolite of α-KG, 2-HG, and downregulating the levels of the activating histone marker of H3K4 methylation (H3K4me3) in the promoter and CNS2 regions of the IL-17 gene (**Figure 2**) (51, 52). PPARγ agonists also downregulate glutathione (GSH) levels, increasing reactive oxygen species (ROS) levels, and downregulating retinoic acid receptor-related orphan receptor γt (RORγt) expression, which facilitates amelioration of Th17-cell-associated immune dysregulation (**Figure 2**) (52). In addition to Gln facilitating the differentiation of Th17 cells, the differentiation of Th17 cells may also promote Gln uptake and Gln metabolism (53). Inducible cAMP early repressor (ICER), transcriptional factor of Th17 cells, promote the expression of GLS1 and the differentiation of Th17 cells by binding with the promoter of GLS1(**Figure 2**) (47). The activation of human CD4^+^ T cells that depend on CD3/CD28 signaling promote GLS expression (46). The stimulation of CD3/CD28 promotes mRNA expression of SLC38A1 and SLC38A2, amino acid transporters that allow Gln to enter cells, as well as relocalizing SLC38A2 from the intracellular reservoirs to the cell surface (53). The activity of GLS and GDH is higher in activated than resting T cells in the mouse spleen, which can be blocked by inhibiting the ERK signal pathway, the downstream of TCR/CD28 signaling (53). Under stimulation of CD3/CD28, Gln deprivation decrease the proliferation and activity of CD4^+^ T cells in both of normoxia and hypoxia (54). Meanwhile, both GLS1 inhibitors BPTES and 968 reduce the secretion of cytokines Th1 and Th17 from CD4^+^ T cells under this conditions (46). Endogenous synthesis of Gln under hypoxic conditions was rate-limiting relative to normoxia where only maximal oxygen consumption rate (OCR) was sensitive to GLS inhibition, and inhibition of GLS or glutamine synthetase (GS) reduced basal and maximal OCR (54). In summary, endogenous synthesis of Gln, closed related with oxygen content, is the key regulatory process in proliferation and glycolysis of CD4^+^ T cells (54).

Inhibition of GLS leads to increased intracellular Gln, decreased Glu, and decreased intracellular levels of a-KG (10). After administration of CB839, an inhibitor of GLS1, Th1 cells showed increased histone methylation, which resulted in decreased expression of PIK3IP1, a regulator of mammalian target of rapamycin complex 1 (mTORC1) signaling, and sensitivity to IL-2 signaling in Th1 cells, activating mTORC1 to promote the differentiation of effector Th1 cells (**Figure 2**) (10). The combination of naïve T-cell activation and rapid Gln uptake depends on SLC1A5 (also known as ASCT2), a deficiency of which reduces the differentiation of Th1 cells *in vivo*, impairs Th1 induction, and attenuates inflammatory T-cell responses (49). The GLS1 splice variant GAC is the major isoform overexpressed in many proliferating lymphocytes (55). Previous studies have demonstrated that GAC is expressed in activated human T cells and that Gln deprivation and GAC inhibition attenuate T cell activation and clonal expansion (46). As a novel GLS1 inhibitor, the recent reported compound 19 (C19) is thought to have a similar or greater ability to inhibit anti-CD3/CD28-induced CD4^+^ T cell proliferation and cytokine production by directly binding to the GAC outside the active site compared to BPTES (55).

It is increasingly evident that Gln is an immunomodulatory nutrient with multiple biological capabilities and metabolic pathways that are crucial in determining the function of different immune subgroups, especially Th17 cells. These studies suggest that Th17 cells are more dependent on Gln metabolism and that Gln metabolism is higher in Th17 cells than in Treg cells. Recently, glutamine-related metabolic pathways, such as the glutamine-glutamate-GSH pathway, glutamine-glutamate-α-KG-2-HG pathway, and glutamine-mTOR signaling, which participate in regulating Th17/Treg cell differentiation, have been identified (56). The discovery of these pathways has made it possible to affect Th17/Treg cell-associated diseases by manipulating or targeting the regulation of Gln metabolism (56). Overall, the metabolism of T cells and Gln catabolism are interdependent and inseparable from each other.

## 4 Glutaminolysis in autoimmune diseases

Under normal conditions, the activity of T cells is controlled by immune tolerance mechanisms that distinguish between autologous and nonautologous components and specifically recognize and react against pathogens (28). When the immune tolerance mechanism is imbalanced, T cells mistakenly generate an immune response against components of the organism itself through the TCR (28). If this response is strong enough to cause inflammation, the function of the tissues may be disrupted. Dysfunction of the body’s tissues caused by autoreactive T cells is called an autoimmune disease (28). As an immunomodulatory nutrient essential for the proliferation and activation of T cells, Gln can also participate in the development of many autoimmune diseases.

### 4.1 Systemic lupus erythematosus

Systemic lupus erythematosus (SLE) is defined as a chronic, recurrent autoimmune disease with recurrent episodes of tissue inflammation and severe multiorgan damage mediated by the body’s autoimmunity (57–59). The pathogenesis of SLE has not been completely understood until now. An imbalance of helper T cells (Th17) and regulatory T cells (Tregs) was proposed to be the underlying pathogenesis of SLE (47). T-cell differentiation and function are regulated by cellular metabolism and Gln catabolism as one of the metabolic features of SLE T cells. Th17 cells in SLE patients are believed to contribute to an inflammatory state in the organs (60–62). Hypoxia-inducible factor 1α (HIF1α) serves as a critical metabolic sensor in Th17 cells, and GLS1 is essential for Th17 differentiation and glycolysis promotion. The inhibition of GLS1 inhibits glycolysis by decreasing HIF1α protein, indicating that the suppression or lack of GLS1 decreases Th17 differentiation and glycolysis *via* the reduction of HIF1α. Lupus in MRL/lpr mice was improved by GLS1 inhibition or deficiency. At the translational level, GLS1 inhibition reduced Th17 differentiation of CD4^+^ T cells in SLE patients *in vitro* (9). Studies have shown that the pathogenesis of SLE is closely linked to inadequate IL-2 production by effector CD4^+^ T cells (63). In lupus-prone mice and SLE patients, GLS2 expression is reduced in CD4^+^ T cells, and GLS2 reduces ROS levels and promotes the ability of CD4^+^ T cells to produce IL-2 by demethylating the IL-2 gene (64). Moreover, overexpression of GLS2 corrected ROS levels and restored IL-2 production by lupus CD4^+^ T cells (64). Tfh cells expanded in SLE, may produce both pathogenic antibodies, and protective antibodies against viral and bacterial pathogens (55). Inhibition of glutaminolysis with the glutamine analogue 6-Diazo-5-oxo-L-norleucine (DON) reduces immune-induced and autoimmune Tfh cellular and humoral responses, selectively targeting pathogenic auto-reactive immune cells while preserving the ability of Tfh to generate protective responses against pathogens (55).

### 4.2 Psoriasis

Psoriasis occurs as a result of a complex interaction between genetic and environmental factors causing DC activation to produce related cytokines such as IFN-α, IFN-β, IL-12, IL-23, IL-6, and TNF-α. These cytokines activate and polarize autoaggressive T-cell subsets, leading to metabolic disorders and serious inflammation-related diseases following immune imbalance of T cells (65–68).

It has been demonstrated that the main IL-17-producing cells in the skin in psoriasis are dermal γδ T cells, which are significantly increased and contribute to disease progression (69, 70). Subcutaneous injection of IL-23 causes psoriasis-like inflammation (71). In contrast, psoriatic mice treated with DON showed reduced keratinocyte overproliferation and leukocyte infiltration, the dorsal skin lesions had reduced epidermal and dermal thickness, and the splenomegaly almost disappeared (50). With the administration of Gln blockade, IL-23-induced γδ T-cell activation can be reversed *in vivo*, and the psoriasis was therefore rescued (50). The expression of genes downstream of IL-17 was suppressed after Gln blockade, and the downregulated genes were enriched in the STAT3-related pathway (50). Previous studies demonstrated that IL-23 induced RORγt (RORC) expression and IL-17 production through activation of STAT3 (72). Therefore, it can be speculated that Gln deprivation may inhibit IL-17 production by suppressing the IL-23-STAT3 pathway (50).

Earlier studies demonstrated that serum α-KG and Glu were abnormally elevated in patients with psoriasis (73–75). In this regard, glutaminolysis may be pivotal in the pathogenesis of psoriasis. More importantly, IL-17A transcription was induced by RORγt (RORC) in Th17 cells (76), which could be enhanced by acetylation of histone H3K9Ac and H3K27Ac (77). The mucosa-associated lymphoid tissue lymphoma translocator protein 1 (MALT1) protease has been shown to play an irreplaceable role in the regulation of GLS1 expression in B-cell lymphomas (78). Consequently, a link between glutaminolysis processes and T cells in psoriasis can be identified. With elevated production of IL-17A by γδ T cells in the serum of psoriasis patients, GLS1-mediated glutaminolysis could promote psoriasis by inducing differentiation of Th17 and γδ Th17 cells (79). MALT1 hydrolase, located upstream of the Gln degradation pathway promoting GLS1-mediated Gln catabolism *via* c-Jun1, promoted epigenetic modification of H3K9Ac and H3K27Ac of the IL17A gene promoter, enhancing the chromatin accessibility of RORγt (RORC), thus exacerbating IL-17A expression and ultimately causing immune peripheral blood imbalance and psoriatic lesions (79).

### 4.3 Multiple sclerosis

Multiple sclerosis (MS) occurs as an autoimmune response to autoantigens mediated by autoreactive T cells secondary to environmentally triggered genetically susceptible hosts (80). The interaction between multiple immunopathological and neuropathological mechanisms in mice with experimental autoimmune encephalomyelitis (EAE), an animal model of MS, results in key pathological features that approximate MS: inflammation, demyelination, axonal loss, and gliosis (81). The CD4^+^ T-cell-specific deletion of ASCT2 significantly suppressed the immune response of Th1 and Th17 cells in a mouse EAE model (81). Activated Th1 and Th17 cells produce inflammatory products and cytokines that disrupt myelin and axons and activate retained microglia, which in turn generate inflammatory cells that attract more inflammatory cells to the central nervous system (CNS) and perpetuate the inflammatory cascade (47). It has been demonstrated that the cAMP response element regulator (CREM) could induce the ICER isoform to promote Th17-cell differentiation, which also enhanced the expression of GLS1, the first enzyme in the Gln catabolic pathway, so that inhibition of GLS1 could improve Th17 differentiation *in vitro* and in mouse EAE (47). Damaged axons in MS lesions are concentrated around the perivascular cuffs of infiltrating cells and macrophages, and axonal injury is strongly connected to glutamate-producing macrophages and microglia (82). Treatment of EAE mice with the Glu antagonist NBQX reduced the extent of axonal damage in active EAE lesions (82). These results suggest that lesion activity and axonal damage in active MS lesions are likely to be caused by Glu excitotoxicity (82).

### 4.4 Systemic sclerosis

As a consequence of dysfunctional differentiation of fibroblasts to myofibroblasts and excessive deposition of extracellular matrix, systemic sclerosis (SSC) is a rare fibrotic autoimmune disease that leads to skin fibrosis (83). Transforming growth factor-β1 (TGF-β1) is an activator that induces the conversion of fibroblasts into myofibroblasts (84). Smad family proteins mediate the signaling of TGF-β family members, and TGF-β1 was recently described to cause upregulation of glutaminase 1 in these cells *via* Smad-dependent pathways and nonclassical pathways (85). The α-KG produced by Gln metabolism serves as an essential substituent for type I collagen and is involved in the composition of the most abundantly expressed extracellular matrix components in fibrosis. Smad activation-mediated TGF-β1 inhibited Gln catabolism in dermal fibroblasts, thereby attenuating the expression of profibrotic markers in SSCs (86).

### 4.5 Crohn’s disease

Crohn’s disease (CD) is an inflammatory bowel disease in which immune imbalance and intestinal mucosal barrier disruption are the main causative factors leading to immune disorders and defective intestinal epithelial barrier function (87). The enhancement of Th1 and Th17 immune responses has critical effects in the pathogenesis of CD (88). The balance of local immunity in intestinal tissues is maintained by regulatory T cells (Tregs) in the gut by suppressing the proliferation and response of other Th cells (89). Treatment with Gln enhanced the integrity of the intestinal barrier in experimental colitis (90). The GLS1-specific inhibitor BPTES significantly inhibited GLS1 expression and increased Gln levels in intestinal tissues (47, 91). Suppressing GLS1 expression could alleviate chronic colitis by maintaining the integrity of the intestinal barrier and Th/Treg homeostasis, improving CD-like colitis (92). Treatment with BPTES downregulated the phosphorylation of the downstream substrates p70S6K and 4E-BP1 of mTORC1 in the intestine of IL-10-/- mice and improved intestinal barrier function and Th/Treg balance to reduce the symptoms of chronic colitis in mice (92).

### 4.6 Rheumatoid arthritis

Rheumatoid arthritis (RA) is a systemic autoimmune disease that results in progressive joint destruction due to the infiltration and proliferation of immune cells in the synovium (93). Under glutamine-containing conditions, the proliferation of RA-FLSs increases after stimulation with the rheumatoid arthritis fibroblast-like synoviocyte (RA-FLS) growth factor PDGF. Administration of GLS1 inhibitors ameliorated inflammatory arthritis in a mouse model of RA by inhibiting FLS proliferation, suggesting the importance of Gln in RA-FLS proliferation (91).

In summary, glutaminolysis is not only a process that generates energy but is also an essential part of immunometabolism that regulates the immune response. The potential therapeutic effects of targeting this pathway are attracting increasing attention.

## 5 Potential diagnostic and therapeutic application of glutaminolysis in autoimmune diseases

Recent studies have shown that Gln catabolism is associated with functions such as immune cell proliferation, cytokine production, and superoxide production (94). Gln metabolism has the potential to be a target for the treatment of autoimmune diseases. The PPARγ agonists rosiglitazone and pioglitazone were recently found to reduce the levels of 2-HG and H3K4me3 in the colon of mice with colitis and to regulate GSH and ROS levels in lamina propria lymphocytes by modulating GLS1/2-HG/H3K4me3 signaling in mouse models of colitis and asthma (52) (**Table 1**). It improved Th17-cell-associated inflammation and reduced IL-17A expression, slowing the progression of colitis and ameliorating autoimmune disease (52). The PPARγ agonist bergenin naturally prevents neutrophil aggregation (95). By modulating the “CDK1(cyclin dependent kinase 1)-APC/C (ubiquitin ligase)-Cdh1 (ubiquitin ligase activator)” signaling pathway after PPARγ activation, bergenin inhibited GLS1-dependent Gln catabolism, thereby inhibiting Th17 differentiation and significantly reducing the number of neutrophils in the BALF of neutrophilic asthmatic mice and the infiltration of inflammatory cells around bronchi in the lungs of mice with neutrophilic asthma (95) (**Table 1**). In contrast, the PPARγ antagonists GW9662 and siPPARγ abolished the inhibitory effects of bergenin on Gln catabolism and Th17 differentiation (95). In addition, the formation of psoriatic lesions can be promoted by GLS1, a downstream target of MALT1 protease, mediating glutaminolysis to promote acetyl coenzyme A-induced differentiation of Th17 and γδ Th17 cells by activating the Il17a promoters histone 3 at the lysine 9 and 27 residues (H3K9Ac and H3K27Ac) (79) (**Table 1**). Additionally, enhanced proliferation of keratin-forming cells and the secretion of trend factors can be further promoted by GLS1-mediated glutaminolysis induced by the IL-17A/MALT1/c-Jun axis to promote lesion formation. Administration of the GLS1-specific inhibitors BPTES and CB-839 reduced keratinocyte proliferation and chemokine production, decreased Th17 and γδ Th17-cell differentiation and epidermal proliferation, and improved the splenomegaly and skin lesions in a psoriasis-like mouse model (79).

**Table 1:** Targets of glutamine metabolism in the regulation of autoimmune diseases.

Diseases	Species	Target	Reference
Colitis	Mice	GLS1, 2-HG, H3K4me3	(52)
Neutrophilic asthma	Mice	GLS1, CDK1-APC/C-Cdh1	(95)
Psoriasis	Human, mice	GLS1, H3K9Ac, H3K27Ac	(79)
Experimental autoimmune encephalomyelitis	Mice	GLS1, ICER	(47)
Systemic lupus erythematosus	Mice	GLS1, HIF1α	(9)
Sjogren’s syndrome	Mice	GLS1	(96)

GLS1, Glutaminase 1; 2-HG, 2-hydroxyglutarate; H3K4me3, H3K4 methylation; ICER, inducible cAMP early repressor; CDK1 cyclin dependent kinase 1; APC/C, ubiquitin ligase; Cdh1, ubiquitin ligase activator; H3K9Ac and H3K27Ac, histone 3 at the lysine 9 and 27 residues; HIF1α, Hypoxia-inducible factor 1α.

PD-1 deficiency increases the comparative levels of certain intermediates and terminal metabolites, such as Glu, N-methylglutamate, N-acetylglutamate (NAG) and ornithine, that participate in Gln metabolism (97). PD-1 agonist treatment improved airway hyperresponsiveness (AHR) in allergic asthma and suppressed lung inflammation in a humanized mouse model by controlling lung ILC2 transcriptional profiles and cytokine production and limiting type 2 innate lymphocyte (ILC2) proliferation through metabolic regulation and reducing ILC2-mediated Th2 cytokine secretion (97).

Treatment of B6 mice with the GLS1 inhibitor BPTES significantly reduced histological scores in the spinal cord of affected animals, lowered the number of CD4^+^ T cells, IL-17A and IFNγ-producing CD4^+^ T cells in the spinal cord, substantially decreased clinical scores and weight loss, and ameliorated experimental autoimmune encephalomyelitis (47). The mechanism is that ICER promotes Gln catabolism by directly binding to the GLS1 promoter in Th17 cells to increase GLS1 expression (47). Treatment with the selective GLS1 inhibitor BPTES reduced glycolysis and ameliorated lupus-like disease and EAE in MRL/lpr mice (9). BPTES treatment of MRL/lpr mice reduced glycolysis and significantly decreased double-negative T cells (CD3^+^CD4^-^CD8^-^ cells), the urinary albumin/creatinine ratio, glomerular nephropathy score, and T-cell infiltration with IL-17A in the kidneys, relieving the EAE disease activity (9). Moreover, BPTES inhibited Th17 differentiation in SLE patients (9). The mechanism is based on a reduction of Th17 differentiation of CD4^+^ T cells by inhibiting GLS1, glycolysis, and CD4^+^ T-cell differentiation *in vitro* by reducing the level of HIF1α protein, a key metabolic sensor in Th17 cells (9) (**Table 1**). Both *in vivo* and *in vitro* experiments have demonstrated that the GLS1 inhibitor BPTES normalized the effector function of CD4^+^ T cells and effectively improved the dysregulation of exocrine glands in Sjogren’s syndrome (SS) (98). Inhibition of GLS1 by BPTES improves glycolysis and oxidative phosphorylation (OXPHOS) in SS-like CD4^+^ T cells and thus slows the progression of SS (98) (**Table 1**).

Therefore, the study of Gln metabolism and its derivatives offers new opportunities for improving autoimmune diseases (96). However, the majority of the subjects are currently limited to animal models of mouse-related diseases with no evidence directly linked to clinical and preclinical applications. Targeted Gln metabolism therapy is still in the initial stage. Future clinical trials evaluating Gln catabolism in human immune cell responses will be useful in determining its function in autoimmune diseases and may be a potential target for the treatment of autoimmune diseases.

## 6 Conclusions and future perspectives

Autoimmune diseases are chronic, recurrent and even fatal conditions caused by deficiencies in the immune system. To date, the etiology and pathogenesis of autoimmune diseases remain elusive. Even though the pathogenesis of autoimmune diseases is not yet fully elucidated, qualitative or quantitative alterations of T cells are undoubtedly central to the regulation of autoimmune diseases. As the most abundant amino acid in the body, Gln is regarded as an immunomodulatory nutrient. The role of Gln in the biological processes of T cells is irreplaceable.

To summarize, the link between Gln catabolism in the pathogenesis of autoimmune diseases in terms of enzymatic activity and inflammation, regulatory mechanisms, and physiological significance *in vivo* remains to be investigated. Breakthroughs in glutaminolysis for diagnostic and therapeutic applications in autoimmune diseases are future research areas, and glutaminolysis may be a new therapeutic strategy for autoimmune diseases.

## Author contributions

XF, NL and XL made data analysis. XF and XL drafted the manuscript. XS and NH revised the manuscript. YL conceived and designed the study. All authors read and approved the final manuscript.

## Funding

The present work was funded by the National Natural Science Foundation of China (Grant No. 82000755) and Natural Science Foundation of Shandong Province (Grant No. ZR2020QH086).

## Conflict of interest

The authors declare that the research was conducted in the absence of any commercial or financial relationships that could be construed as a potential conflict of interest.

## Publisher’s note

All claims expressed in this article are solely those of the authors and do not necessarily represent those of their affiliated organizations, or those of the publisher, the editors and the reviewers. Any product that may be evaluated in this article, or claim that may be made by its manufacturer, is not guaranteed or endorsed by the publisher.
